# Supplementary material for: The Effect of an Innovative Combination of Bergamot Polyphenolic Fraction and Cynara cardunculus L. Extract on Weight Gain Reduction and Fat Browning in Obese Mice
Source: Int J Mol Sci. 2023 Dec 22;25(1):191. doi: 10.3390/ijms25010191 (PMC10779365; doi:10.3390/ijms25010191)
Supplement: Supplementary file 1 [file ijms-25-00191-s001.zip › ijms-2762595-supplementary.pdf]

SAMPLE REPORT

Acquisition Date/Time	24-May-23 15:06:03
Acquisition Method	Cynaropicrin_06_2021
Chromera Version	4.2.0.6415
Dilution Factor	1004
Report Date/Time	07-Jun-23 13:37:37
Sample Name	HPLC Lot. 0605-23 Cynaropicrin
Vial Number	87

HPLC Lot. 0605-23 Cynaropicrin : 205:11:400:1 : 1

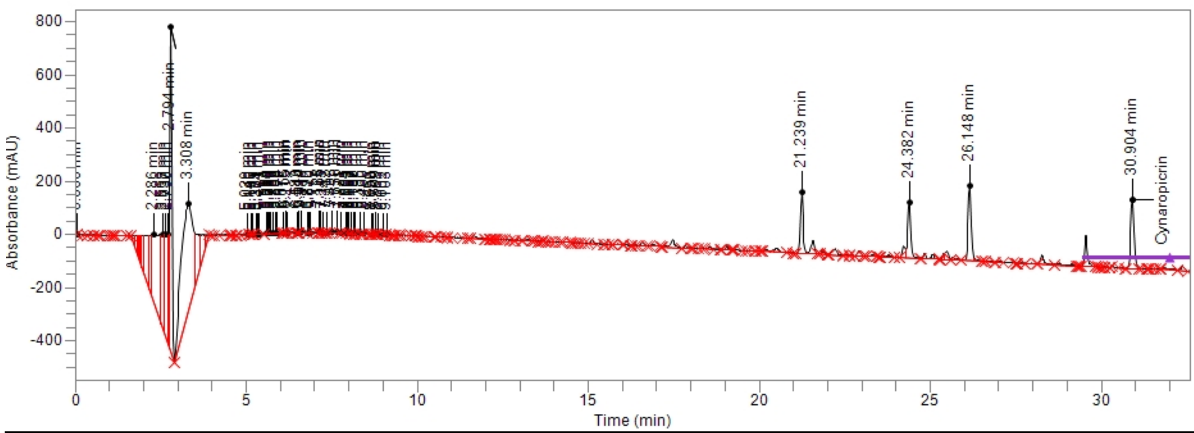

Peak #	Time	Component Name	Area	Height	Final Amount	Units
230	30.904	Cynaropicrin	1,782,352.0	258,390.3	69,654.4808	ppm
**Total**			1,782,352.0		69,654.4808	

SAMPLE REPORT

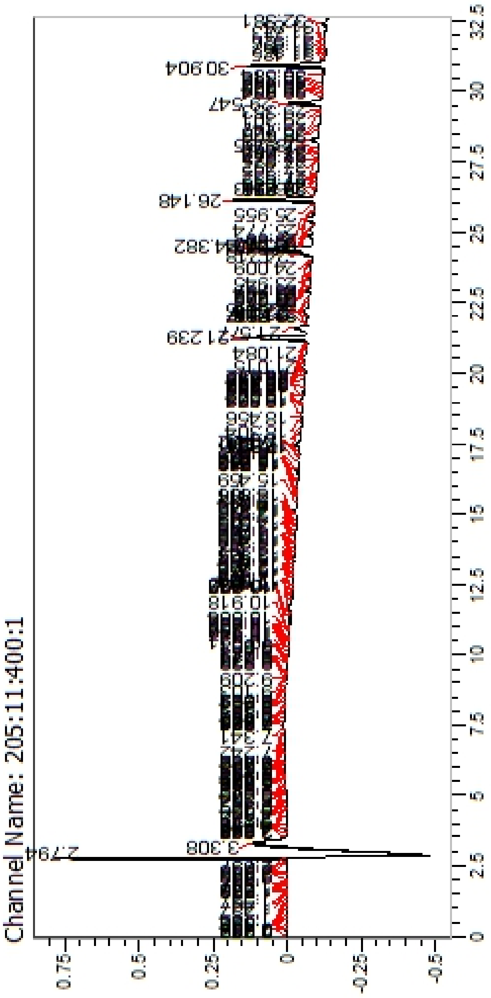

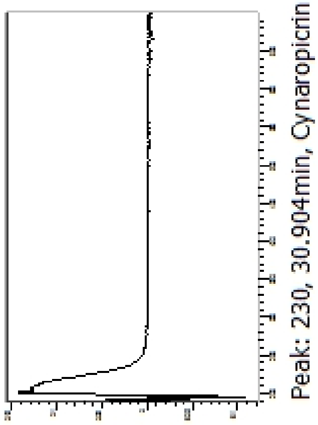

SAMPLE REPORT

Approved:

Signature: ________________ Name: ________________ Date: ______________

SAMPLE REPORT

Acquisition Date/Time	26-May-23 07:59:27
Acquisition Method	Flavonoids ca. on Naringin_march22
Chromera Version	4.2.0.6415
Dilution Factor	1004
Report Date/Time	07-Jun-23 14:15:17
Sample Name	HPLC Lot 0605-23 polyph
Vial Number	87

HPLC Lot 0605-23 polyph : 284:10:400:10 : 1

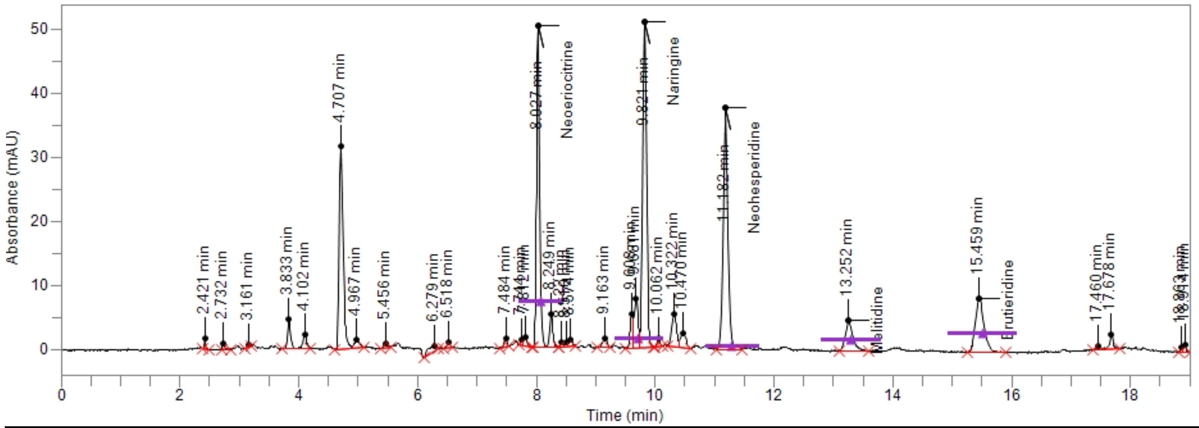

Peak #	Time	Component Name	Area	Height	Final Amount	Units
28	15.459	Brutieridine	87,310.3	8,440.0	23,826.9243	ppm
27	13.252	Melitidine	44,069.9	4,713.8	13,917.5475	ppm
22	9.821	Naringine	221,646.7	50,739.4	54,614.0003	ppm
14	8.027	Neoeriocitrine	179,832.7	49,938.1	45,031.2716	ppm
26	11.182	Neohesperidine	219,459.6	37,626.2	54,112.5642	ppm
**Total**			752,319.3		191,502.3079	

SAMPLE REPORT

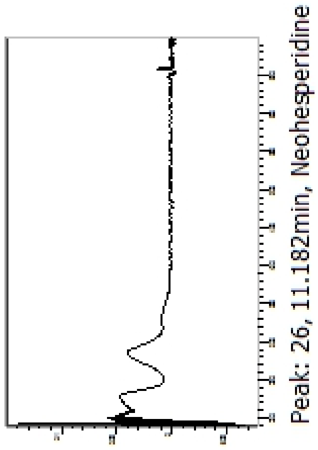

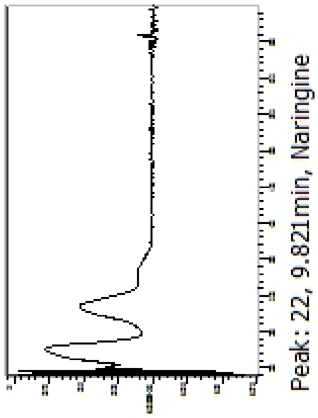

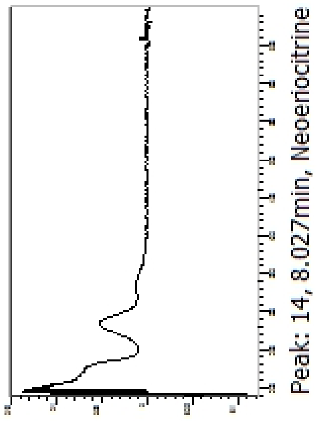

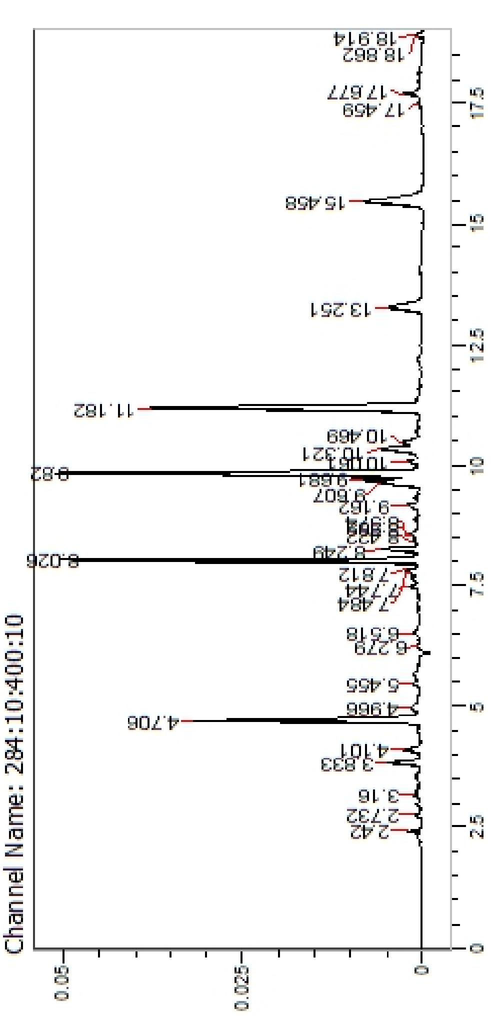

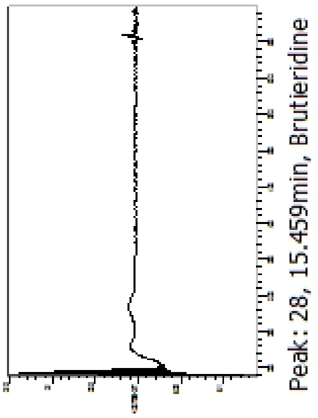

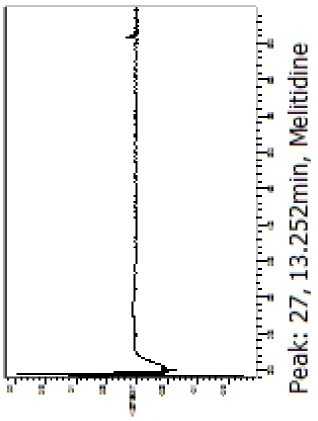

SAMPLE REPORT

Approved:

Signature: ______________ Name: ______________ Date: ____________

Bergacyn® FF

SPECIFICATION SHEET

**ID Code**	**BCGESTPF**	
**Trade name**	**Bergacyn® FF**	
**DESCRIPTION**	**SPECIFICATIONS**	
Botanical Source	Citrus Bergamia Risso et Poit, and Cynara Cardunculus	
Family	Rutaceae (bergamot), and Asteraceae (artichoke)	
Country of Origin	Calabria, Italy	
Part Used	Fruit (bergamot) and leaf (artichoke)	
**ORGANOLEPTIC CHARACTERISTICS**	**SPECIFICATIONS**	
Colour	Gold-green Powder	visual (CQ-MO-148)
Odour	Aromatic	visual (CQ-MO-148)
Flavour	Characteristic of bergamot	sensory (CQ-MO-148)
**CHEMICAL CHARACTERISTICS**	**SPECIFICATIONS**	**METHOD**
pH	3.0 – 5.0	IM (0.5% in water at 25°C)
Average Mesh Size	Pass 60 mesh	Sieve: (CQ-MO-023)
Tapped Density	40 - 70 g/100mL	PT CHIM 65 rev 0 2011
Moisture Content	< 8.0%	ISTISAN 96/34, pag 7
Solubility in 40°C H_2O	Partial	visual: (CQ-MO-148)
Solubility in 50% H_2O + EtOH	Partial	visual: (CQ-MO-148)
Organic Solvent Residue	Complies with Dir. 2009/32/CE	GC: (CQ-MO-168)
Pesticides Residue	Complies with Reg. UE 839/2008	PT CHIM 69rev 02 011
**SINGLE COMPONENT MINIMUM TRESHOLD CONCENTRATION**		
BERGAMOT POLYPHENOLS (Neoeriocitrin, Naringin, Neohesperidin, Melitidin, Brutieridin)	9.5 %	
CYNARA CARDUNCULUS (Cynaropicrin)	5.0 %	
**HEAVY METALS**	**UNIT**	**RANGE**
Arsenic	mg/kg	<1.0
Lead	mg/kg	<0.5
Cadmium	mg/kg	<0.5
Mercury	mg/kg	<0.1
**MICROBIOLOGICAL EVALUATION**		
Aerobic Plate Count	<10,000 CFU/g	ISO 4833-1:2013
Yeast and Mold Count	<1,000 CFU/g	ISO 21527-1:2008
E. Coli	Negative	ISO 16694-2:2001
Coliform	Negative	ISO 4832:2006
Salmonella	Negative	UNI EN ISO 6579:2000
Staphylococcus Aureus	Negative	UNI EN ISO 6888-2:2004
Streptococci	Negative	PT BAT26 rev0 02012
**PRODUCT TREATMENT**		
Drying Method	**Spray dry**	
The product is mixed with 50% Bergacyn® + 50% micronized Bergamot Fibers and Albedo Powder	**Micronized**	

Herbal &Antioxidant Derivatives S.r.l. (H&AD S.r.l)
Headquarters: **Località Chiusi - 89032 BIANCO (RC), ITALY**
Tel. +39 0964 913405 E-mail: info@head-sa.com *Website:* http://www.head-sa.com/
CF/PI- 02543180802

Bergacyn® FF

NUTRITIONAL FACTS

**PRODUCT NAME:**	**Bergacyn® FF**
**IDENTIFICATION CODE:**	**BCGESTPF**
**NUTRIENTS**	**AMOUNT per 100g**
Calorific Value (kcal)	**300 - 450**
Fat (%)	**< 1.0**
Ash (%)	**< 7.0**
Total Carbohydrates (%)	**> 80.0**
Protein (%)	**< 2.5**
Water (%)	**< 8.0**

RECOMMENDED STORAGE CONDITIONS:

Store in a fresh and dry place, repaired from light and humidity sources.

BEST BEFORE:

36 months under the previously mentioned conditions and in its original packaging.

PACKAGING:

20 Kg fiber drums.

Giuseppe Trunfio
H&ad srl analytical chemistry & processes
***E-mail :** g.trunfio@head-sa.com*

Herbal &Antioxidant Derivatives S.r.l. (H&AD S.r.l)
Headquarters: **Località Chiusi - 89032 BIANCO (RC), ITALY**
Tel. +39 0964 913405 E-mail: info@head-sa.com *Website: http://www.head-sa.com/*
CF/PI- 02543180802
